# Psychometric properties of the Japanese translation of the De Jong Gierveld Loneliness Scale for young and older adults

**DOI:** 10.3389/fpsyg.2025.1542961

**Published:** 2025-06-27

**Authors:** Honami Arai, Sho Okawa

**Affiliations:** ^1^Center for Research on Counseling and Support Services, University of Tokyo, Tokyo, Japan; ^2^Department of Life Sciences, University of Tokyo, Tokyo, Japan

**Keywords:** DJGLS, psychometric properties, social loneliness, emotional loneliness, young adult, older adult

## Abstract

**Objective:**

The De Jong Gierveld Loneliness Scale (DJGLS) has been widely used to measure two types of loneliness: social and emotional loneliness. However, no Japanese translation has been developed. Furthermore, the characteristics of social and emotional loneliness in the Japanese population remain unclear. This study examined the psychometric properties of a Japanese translation of the DJGLS.

**Methods:**

A total of 1,000 participants (500 young and older adults each) completed online questionnaires, which included items on loneliness, depression, and social isolation.

**Results:**

Confirmatory factor analyses revealed a two-correlated factor structure for the Japanese translation of the DJGLS in both young and older adults. McDonald’s omega coefficient was high for both social and emotional loneliness. The emotional loneliness subscale was moderately correlated with general loneliness and depression. Furthermore, the social loneliness subscale was moderately correlated with general loneliness and social isolation. Depression was more strongly correlated with emotional loneliness than with social loneliness. Item response theory demonstrated the characteristics of each item in the Japanese translation of the DJGLS.

**Conclusion:**

These results support the validity and reliability of the DJGLS among young and older Japanese adults.

## 1 Introduction

Loneliness, a state of negative affect, occurs when a person’s social connections are insufficient in quantity or quality (Perlman and Peplau, 1981). Although a prevalent negative state across all ages worldwide, loneliness is particularly prevalent among older adults (Yang and Victor, 2011). It is a risk factor for poor social relationships, mental health problems, and suicidal ideation (Stravynski and Boyer, 2001; Victor and Yang, 2012). In Japan, loneliness became more salient during the COVID-19 pandemic, even among younger individuals, with 42.7–50.3% of those under the age of 60 being categorized as lonely (Stickley and Ueda, 2022).

Loneliness can be categorized into two types: social and emotional loneliness (Weiss, 1973). According to Walsh et al. (2025), social loneliness relates to perceived social network deficits while emotional loneliness refers to the perceived lack of intimate connections. Previous studies have reported a moderate-to-low correlation between social and emotional loneliness (Russell et al., 1984; Wolters et al., 2023), indicating they represent related but distinct constructs. In addition, each dimension functions differently. Social loneliness is more strongly associated with social isolation, whereas emotional loneliness showed stronger association with psychological distress, including social anxiety and depression (Wolters et al., 2023). Furthermore, emotional loneliness was significantly correlated with the presence of a partner; however, no correlation was observed in social loneliness (Green et al., 2001). As Walsh et al. (2025) demonstrate, emotional loneliness appears to be a more severe typology with substantial overlap with psychological pathology, while social loneliness reflects deficits in social connectedness and social support. This distinction has been replicated in Japanese populations, where emotional loneliness was found to predict dementia risk while social loneliness showed no such association (Shibata et al., 2021). These findings collectively support the importance of separately assessing emotional and social loneliness as distinct constructs.

The De Jong Gierveld Loneliness Scale (DJGLS) measures both social and emotional loneliness. It comprises 11 items: five positive-worded items for social loneliness and six negative-worded items for emotional loneliness (De Jong Gierveld and van Tilburg, 2023). Both types of loneliness in the DJGLS are associated with another loneliness scale and negative affect in young and older adults (Green et al., 2001). The DJGLS has been translated and validated in several countries, including Italy, Spain, and Iran (Alsubheen et al., 2023). Although a short version has been translated into Japanese, the original 11-item version has not yet been translated or validated in Japan (De Jong Gierveld and van Tilburg, 2010). Hence, development of a Japanese translation of the 11-item DJGLS can be beneficial to comprehensively capture the characteristics of loneliness in the Japanese population.

Cultural factors in Japan may influence widespread loneliness. Perception of relational mobility is a cultural factor associated with loneliness in the Japanese population. Japan is categorized as a country with the lowest relational mobility, which implies that individuals tend to remain in the same social group and are less likely to change groups (Thomson et al., 2018). Recent research has also found that perceptions of social rigidity in one’s environment serve as a major correlate of loneliness across the Japanese population (Badman et al., 2022). Low relational mobility has been associated with loneliness among the Japanese (Thomson et al., 2018). This unique cultural characteristic implies that Japanese translations of measures may have different psychometric properties than those of the original and other translated measures of loneliness.

This study aimed to translate the 11-item DJGLS into Japanese and examine its psychometric properties in young and older adults. We targeted both young and older adults owing to the U-shaped age distribution of loneliness (Shah and Househ, 2023). First, the factor structure of the Japanese translation of the DJGLS was examined. We hypothesized that the Japanese translation would have a two-correlated factor structure that comprised social and emotional loneliness. Second, we separately calculated the reliability of the Japanese translation for young and older adults. We hypothesized that McDonald’s omega (*ω*) coefficient would be ≥0.70 for both. Third, we assessed the convergent validity based on the correlation coefficients between the subscales of the DJGLS and general loneliness, social isolation, depression, and presence of a partner. We hypothesized that both social and emotional loneliness would have significant positive correlations with general loneliness, depression, and social isolation. Fourth, we compared the correlation coefficient of each subscale with presence of a partner and depression to assess the discriminant validity of social and emotional loneliness. We hypothesized that emotional loneliness would have a stronger correlation with presence of a partner and depression compared with social loneliness. Finally, we exploratorily examined the characteristics of each item in the DJGLS via the item response theory (IRT) approach. We examined the psychometric properties of the Japanese translation of the DJGLS separately for young and older adults to demonstrate its usability for two different age ranges in the Japanese population.

## 2 Methods

### 2.1 Participants

We recruited 500 young and older adults each (age range: 18–29 and 65 years or older, respectively) through Cross Marketing, a nationwide online research company. Cross Marketing was selected to enable geographically diverse recruitment from across Japan, drawing from their panel of approximately 12,850,000 registered individuals nationwide. This study employed panel sampling methodology through Cross Marketing’s established participant database. Participants received points that could be used for shopping as compensation. Inclusion criteria were that participants were Japanese nationals and aged 18–29 years and 65 years or older for the young adult and older adult sample, respectively. No additional exclusion criteria were applied. After removing participants with response biases (23 young adults and 7 older adults), the final sample consisted of 477 young adults and 493 older adults. Table 1 presents the participants’ characteristics. Sample size was set based on the recommended sample size to conduct the IRT. Sample size for both young and older adults was sufficient to conduct the Rasch model, which recommended 400 or more participants (Kolen and Brennan, 2004). Additionally, the sample size meets the requirements for confirmatory factor analysis and provides an adequate sample size to detect small to moderate correlation coefficients (Bujang and Baharum, 2016; Hair et al., 2010). This study was approved by the institutional review board of the University of Tokyo (#23–480). Informed consent was obtained from all the participants before they completed the questionnaire. After the participants read the informed consent form, they were asked to choose to agree or disagree to participate. Only those who agreed to participate were provided access to the questionnaires. Survey responses were anonymous, and no minors were involved in this study. Data were collected from February 9 to 13, 2024. The study protocol was registered in the Open Science Framework^1^^,^ ^2^.

**Table 1:** Participants’ characteristics.

Characteristics	Young adult (*n* = 477)	Older adult (*n* = 492)
Age: mean (SD)	25.14 (3.17)	70.82 (4.39)
Gender: *n* (%)		
Female	359 (75.26)	113 (22.97)
Male	114 (23.90)	379 (77.03)
Others	4 (0.84)	0
Presence of a partner: *n* (%)	394 (80.08)	203 (42.56)
Employment status: *n* (%)		
Unemployed	67 (14.05)	292 (59.35)
Part-time	92 (19.29)	79 (16.06)
Full-time	209 (42.81)	96 (19.51)
Student	90 (18.87)	0
Others	19 (3.98)	25 (5.08)
Education: *n* (%)		
Junior high school	20 (4.19)	2 (0.41)
High school	112 (23.48)	145 (29.47)
University	319 (66.88)	315 (64.02)
Graduate school	21 (4.40)	28 (5.69)
Others	5 (1.05)	2 (0.41)
Communication with friends*: *n* (%)		
In person	400 (83.86)	427 (86.79)
By phone	312 (65.41)	364 (73.89)
By SNS	374 (78.41)	264 (53.66)
By email	203 (42.56)	329 (66.87)

*Communication with friends indicates the frequency and percentage of individuals who communicate with their friends at least once a month.

### 2.2 Measures

#### 2.2.1 Demographic questionnaire

The demographic questionnaire enquired about the participants’ age, gender, presence of a partner, employment status, education, and frequency of communication with friends.

#### 2.2.2 University of California, Los Angeles Loneliness Scale (UCLA-LS)

The UCLA-LS was used to measure general loneliness and comprised 20 items scored on a 4-point Likert scale (Russell, 1996). The Japanese translation of the UCLA-LS had good internal consistency (Cronbach’s *α* = 0.92; Masuda et al., 2012). Its construct validity was supported by a significant positive and negative correlation with the depression scale and self-perceived health, respectively (Masuda et al., 2012). Higher scores indicated higher levels of general loneliness. The reliability of the UCLA-LS for this study was adequate for both young (McDonald’s *ω* = 0.89) and older (McDonald’s *ω* = 0.93) adults.

#### 2.2.3 Patient Health Questionnaire-9 (PHQ-9)

The PHQ-9 was used to measure depressive symptoms and asked participants to rate how frequently they had been bothered by depressive symptoms in the past 2 weeks (Spitzer et al., 1999). It comprised nine items rated from 0 (not at all) to 3 (nearly every day). The validity of the Japanese translation was supported by its diagnostic accuracy to detect depression, with a sensitivity of 0.86 and specificity of 0.85 (Inagaki et al., 2013). Higher scores indicated more severe depressive symptoms. The reliability of the PHQ-9 for this study was adequate for both young (McDonald’s *ω* = 0.93) and older (McDonald’s *ω* = 0.91) adults.

#### 2.2.4 Lubben Social Network Scale (LSNS)

The LSNS was used to measure social isolation on a 6-point Likert scale (Lubben, 1988). It comprised six items that assessed social support from family members (three items) and friends (three items). The Japanese translation had good internal consistency (Cronbach’s *α* = 0.82), test–retest reliability (*r* = 0.92), and concurrent validity (Kurimoto et al., 2011). We reversed the LSNS scores to clarify the interpretation of the association with loneliness. In the LSNS, higher scores indicated higher social isolation. The reliability of the LSNS for this study was adequate for both young (McDonald’s *ω* = 0.88) and older (McDonald’s *ω* = 0.85) adults.

#### 2.2.5 11-item De Jong Gierveld Loneliness Scale (DJGLS)

The DJGLS was used to measure loneliness and comprised 11 items scored on a 5-point Likert scale (De Jong-Gierveld and Kamphuls, 1985). Of these, six negatively phrased and five positively phrased items measured emotional loneliness (EL) and social loneliness (SL), respectively. We followed the DJGLS manual and converted the EL and SL scores into binary scores (De Jong Gierveld and van Tilburg, 2023). Neutral and positive answers were scored as 1 and negative answers as 0 for negatively phrased items. Conversely, neutral and negative answers were scored as 1 and positive answers as 0 for positively phrased items. Higher scores indicated higher EL and SL. The DJGLS has been translated into several languages, and its validity and reliability have been supported (Alsubheen et al., 2023).

We referred to the COSMIN checklist manual to develop a Japanese translation of the DJGLS (Mokkink et al., 2012). Prior to the translation procedure, we obtained permission from the original author to develop a Japanese translation. Items were translated by two different research teams and combined during the final translation procedure. First, two bilingual clinical psychologists, from a research team, who had experience living in English-speaking countries separately translated the English items into Japanese. Another research team, led by a psychiatrist, also translated the English items into Japanese. Second, independent translators from a translation agency separately back-translated the Japanese-translated items into English for both teams. Third, a researcher with expertise in loneliness and involved in the preparation of the original DJGLS manual checked the back-translation to assess whether the original intention was reflected. The researcher separately checked the back-translation for both teams. Fourth, the two research teams came together and discussed the Japanese translation based on comments from the researcher. The teams had difficulty arriving at a consensus for one item. Therefore, we asked for comments on the two back-translations from the researcher, who checked them and confirmed the final version of the Japanese-translated items in the DJGLS.

### 2.3 Statistical analysis

We examined the factor structure, reliability, convergent validity, discriminant validity, and item characteristics of the Japanese version of the DJGLS. All analyses were conducted separately for the young and older adults samples. First, we conducted a confirmatory factor analysis (CFA) using a weighted least squares with means and variance adjustment (WLSMV) estimator to confirm the two-correlated factor structure of the DJGLS^3^. WLSMV estimator was selected because it is specifically designed for analyzing binary and categorical data, which is appropriate given the binary nature of the DJGLS items. We evaluated the model via the comparative fit index (CFI), root mean square error of approximation (RMSEA), and standardized root mean square residual (SRMR) indices. We set the following criteria to determine the model’s acceptability: CFI > 0.90 and >0.95 as acceptable and good, RMSEA <0.08 and <0.06 as acceptable and good, SRMR <0.08 and <0.05 as acceptable and good, respectively (Hu and Bentler, 1999; Schumacker and Lomax, 2010). In addition to model fit indices, we considered factor loadings and model interpretability to determine model acceptability (McNeish et al., 2018). Second, we estimated McDonald’s omega subscale separately for the social and emotional subscales of the DJGLS. Third, we conducted Spearman’s correlation analysis to determine the correlation between the subscales of the DJGLS and UCLA-LS, PHQ-9, and LSNS. We used point-biserial correlations to examine the correlation between the DJGLS subscales and presence of a partner. Correlations were interpreted as negligible (0 to ±0.20), weak (±0.21 to ±0.40), moderate (±0.41 to ±0.60), strong (±0.61 to ±0.80), or very strong (±0.81 to ±1.00; Prion and Haerling, 2014). Fourth, we used the CORTESTI function in STATA to compare the correlation between the subscales of the DJGLS and PHQ-9 and LSNS (Caci, 2000). Finally, we used the Rasch model of the IRT to examine the item characteristics of the DJGLS. Two separate IRT analyses were conducted for SL and EL. Since the IRT can be used for unidimensional measures, we checked the unidimensionality of SL and EL before we estimated the IRT parameters. We estimated the difficulty parameters, Weighted Mean Square (WMS) Infit, and Unweighted Mean Square (UMS) Outfit for each item. The difficulty parameter indicated the trait level (theta) where 50% of the population endorsed score 1. Higher difficulty indicated that a higher trait level was required to endorse item score 1 (Tesio et al., 2024). WMS and UMS were fit statistics calculated by the squared mean difference between the observed and expected responses in the Rasch model. A result of 2.0 or more indicated distortion of the measurement system (Linacre, 2002). We created an item characteristic curve for each item based on their difficulty parameters. The CFA was conducted using Mplus version 8, McDonald’s omega, Spearman’s correlation, and CORTESTI were conducted using Stata 16, and the Rasch model of the IRT using jMetrik 4.1.1. Item information function curve figures were created via the Matplotlib 3.8.3 library in Python.

## 3 Results

### 3.1 Factor structure of the DJGLS

We conducted a CFA to examine whether the Japanese translation of the DJGLS had the two-correlated factor structure reported in the original DJGLS in Western countries. For the young adult sample, the model fit indices were inadequate (CFI = 0.94, RMSEA = 0.10, SRMR = 0.11). We checked the model’s modification indices to identify the problems and attain a better-fitting model. The model modification index of the path from the social loneliness factor to item 5 in the DJGLS was high (Modification index = 156.98). Accordingly, we estimated a two-correlated factor model with cross-loading of item 5 on both factors. This was significant for both emotional (factor loading = 0.77) and social loneliness (factor loading = −0.47). Furthermore, the model fit improved to an acceptable level (CFI = 0.98, RMSEA = 0.05, SRMR = 0.06). However, the cross-loading of item 5 could indicate an unclear distinction between the two factors and cause interpretation difficulties in the future. Therefore, we also assessed a two-correlated factor model without item 5, which yielded an acceptable model fit (CFI = 0.99, RMSEA = 0.04, SRMR = 0.05). To maintain a clear and distinct view of the two factors, we accepted the two-correlated factor model without item 5 for the young adult sample. For the older adult sample, we accepted a two-correlated factor model as the model fit indices were acceptable (CFI = 0.99, RMSEA = 0.04, SRMR = 0.06). Table 2 presents the factor loadings of each item in the accepted models for young and older adults. The factor loadings ranged from 0.67 to 0.97 for each factor in the young and older adult samples.

**Table 2:** Factor loadings of the two-correlated models for young and older adults.

Item	Young adults	Older adults
	Factor loading	Factor loading
Emotional loneliness		
Item 2	0.81	0.86
Item 3	0.83	0.80
Item 5	–	0.70
Item 6	0.72	0.86
Item 9	0.83	0.81
Item 10	0.85	0.79
Social loneliness		
Item 1	0.86	0.67
Item 4	0.95	0.86
Item 7	0.86	0.95
Item 8	0.92	0.97
Item 11	0.88	0.84
Latent factor covariance		
Emotional loneliness – Social loneliness	0.23	0.43
Model fit		
CFI	0.99	0.99
RMSEA	0.04	0.04
SRMR	0.05	0.06

### 3.2 Reliability

McDonald’s *ω* coefficients were estimated for each factor separately for young and older adults and were 0.80 and 0.81 for the emotional loneliness factor and 0.86 and 0.83 for the social loneliness factor, respectively.

### 3.3 Convergent and discriminant validity

Table 3 presents the means and standard deviations of each factor and scale for young and older adults. We estimated Spearman’s correlation coefficients between emotional/social loneliness and the UCLA-LS, PHQ-9, and LSNS. For presence of a partner, point-biserial correlations were used to estimate correlation coefficients. Table 4 presents the correlation coefficients. Emotional loneliness was moderately correlated with the UCLA-LS and PHQ-9 and social loneliness was moderately correlated with the UCLA-LS and LSNS in both the samples. Other variables were weakly or less than weakly correlated with social and emotional loneliness in both samples. Correlations between emotional loneliness and the PHQ-9 scores were significantly stronger in young [*t*(474) = 5.09, *p* < 0.001] and older adults [*t*(489) = 4.90, *p* < 0.001] than those between social loneliness and the PHQ-9 scores. Correlations between social loneliness and presence of a partner were significantly stronger than those between emotional loneliness and presence of a partner among young adults [*t*(474) = −2.48, *p* = 0.013]. No significant differences were observed between the correlation coefficients of social and emotional loneliness and presence of partner in older adults [*t*(489) = −1.00, *p* = 0.317].

**Table 3:** Mean and SD for each scale for young and older adults.

Scale	Young adults		Older adults	
	Mean	SD	Mean	SD
UCLA LS	48.68	11.42	44.49	10.66
PHQ-9	7.79	7.09	3.88	4.89
LSNS	10.27	6.75	10.95	6.45
EL	2.34	1.85	1.97	1.98
SL	3.53	1.80	3.61	1.69

UCLA-LS = University of California, Los Angeles Loneliness Scale, PHQ-9 = Patient Health Questionnaire-9, LSNS = Lubben Social Network Scale, EL = Emotional Loneliness, SL = Social Loneliness.

**Table 4:** Correlation coefficients between emotional/social loneliness and the UCLA-LS, PHQ-9, LSNS, and presence of a partner.

Scale	Young adults		Older adults	
	EL	SL	EL	SL
SL	0.13**		0.23***	
UCLA-LS	0.54***	0.49***	0.48***	0.63***
PHQ-9	0.45***	0.16***	0.44***	0.18***
LSNS	0.17***	0.52***	0.19***	0.55***
Partner	−0.10*	−0.25***	−0.02	−0.08

EL = Emotional Loneliness, SL = Social Loneliness, UCLA-LS = University of California, Los Angeles Loneliness Scale, PHQ-9 = Patient Health Questionnaire-9, LSNS = Lubben Social Network Scale.

***p < 0.001, **p < 0.01, *p < 0.05.

### 3.4 Item characteristics

We conducted a principal component analysis to confirm the unidimensionality of each factor in the DJGLS. Since the first component explained more than 20% of each factor (young adults: EL = 56.01%, SL = 65.13%; older adults: EL = 51.78%, SL = 60.53%), we assumed that all the factors met the assumption to conduct IRTs (Nguyen et al., 2014).

**Figure 1:**
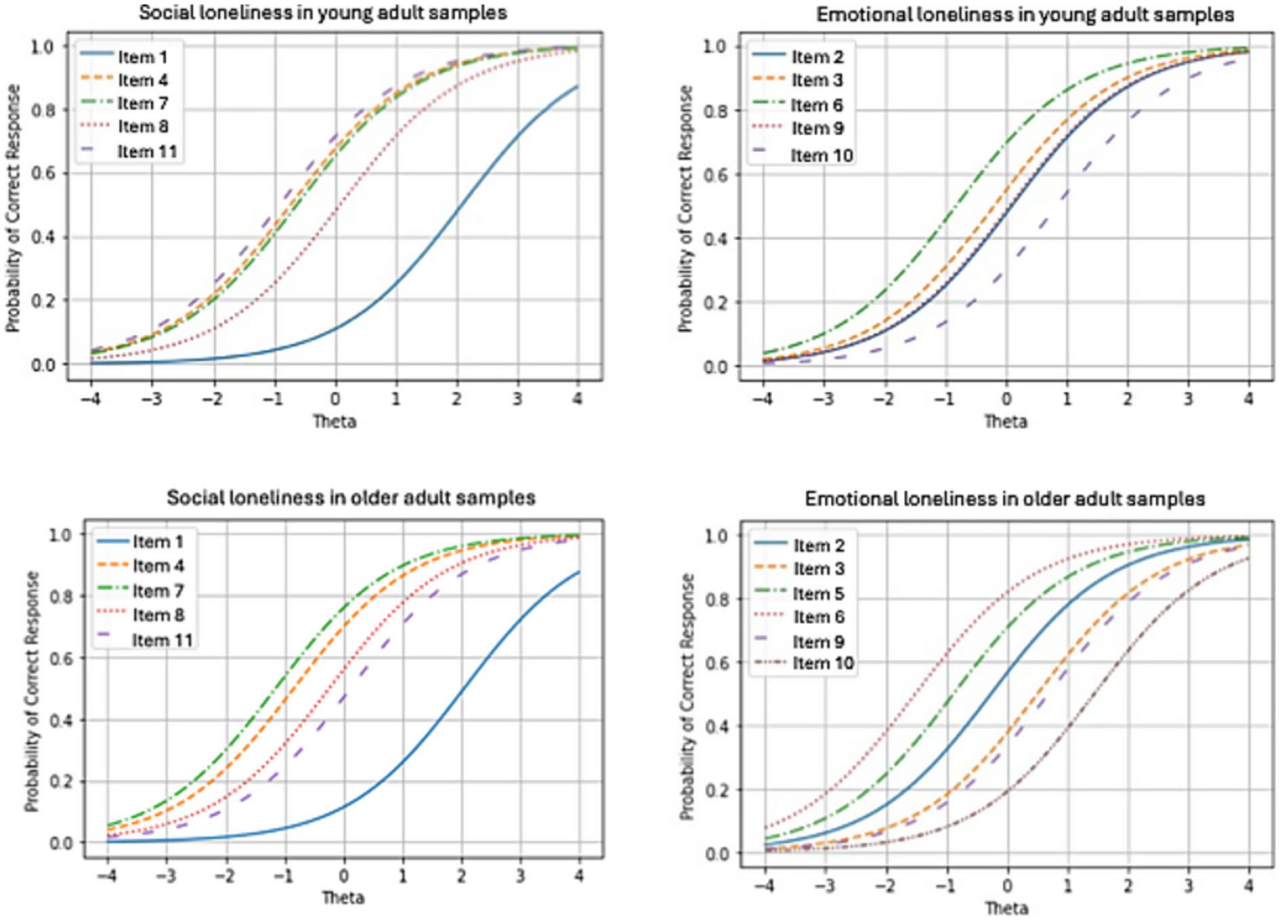
Item characteristic curve for each item in young and older adults.

We used the Rasch model to estimate the difficulty parameters, WMS, and UMS, for emotional and social loneliness factors in both samples. The UMS of item 1 in the older adult sample exceeded 2 (item 1: difficulty = 1.82, WMS = 1.42, UMS = 2.47). Since UMS was outlier-sensitive, we treated individuals with high UMS as missing data and fitted the Rasch model for the older adult sample. Table 5 presents the parameters of the Rasch model. Figure 1 illustrates the characteristic curves for each item of the DJGLS for young and older adults. In the young and older adult samples, difficulty parameters ranged from −0.82 to 0.84 and −0.89 to 2.11 and −1.51 to 1.45 and −1.14 to 2.06 for the emotional and social loneliness factors, respectively. All the WMS and UMS were within the range of ±2 in the final models.

**Table 5:** Rasch model of the IRT parameters.

Item	Young adults			Older adults		
	Difficulty	WMS	UMS	Difficulty	WMS	UMS
Emotional loneliness						
Item 2	0.10	1.02	1.02	−0.26	0.95	0.98
Item 3	−0.19	0.97	0.97	0.51	0.98	1.00
Item 5	–	–	–	−0.88	1.14	1.16
Item 6	−0.82	1.16	1.22	−1.51	0.94	0.90
Item 9	0.06	0.92	0.89	0.69	0.97	0.89
Item 10	0.84	0.94	0.90	1.45	1.00	1.16
Social loneliness*						
Item 1	2.11	1.06	1.07	2.06	1.32	1.76
Item 4	−0.71	0.84	0.70	−0.83	0.99	0.81
Item 7	−0.61	1.12	1.12	−1.14	0.85	0.70
Item 8	0.10	0.90	0.87	−0.23	0.74	0.64
Item 11	−0.89	1.10	1.19	0.13	1.08	1.11

WMS = Weighted Mean Square, UMS = Unweighted Mean Square.

*Eight samples of social loneliness in older adults were treated as missing values owing to a high misfit (UMS = 6.72).

## 4 Discussion

This study aimed to translate the 11-item DJGLS into Japanese and examine its psychometric properties in young and older adults. We conducted a CFA to examine whether the two-correlated factor structure of the DJGLS fit the Japanese population. In addition, we examined its reliability based on McDonald’s omega coefficients. Spearman’s and point-biserial correlations were used to examine the convergent and discriminant validity of the Japanese translation, respectively. We also conducted an IRT analysis to examine the characteristics of each item.

As hypothesized, the Japanese translation of the DJGLS yielded a good fit with adequate factor loadings for the two-correlated factor structure for both young and older adults, which was consistent with the original DJGLS (De Jong Gierveld and van Tilburg, 2023). Although all 11 items loading on emotional and social loneliness were consistent with the original DJGLS in older adults, the model showed poor fit in young adults. The model modification index suggested a negative loading of item 5 (“I miss the pleasure of the company of others”) on social loneliness, in addition to a positive loading on emotional loneliness. The cross-loading of item 5 may be due to the frequent use of the internet and social media among young adults. One study suggested that the frequency of internet use was positively associated with emotional loneliness and negatively associated with social loneliness (Moody, 2001). Approximately 78.41% of young adults in this study used social media to communicate with their friends. Conversely, 53.66% of older adults used social media to communicate with their friends. Internet and social media use could increase the quantity although not the quality of social connections, which may lead item 5 to load negatively on social loneliness yet positively on emotional loneliness for younger adults. To ensure that the Japanese translation captured two separate constructs of loneliness, we removed item 5 and accepted 10 items of the two-correlated factor structure for younger adults.

The second hypothesis that McDonald’s *ω* coefficient would be ≥0.70 in both young and older adults’ reports was supported. McDonald’s ω coefficients of emotional and social loneliness ranged from 0.80–0.86, which indicated sufficient reliability (McNeish, 2018). Thus, this study supported the reliability of the subscales of the Japanese translation of the DJGLS.

The third hypothesis that social and emotional loneliness would have significant positive correlations with general loneliness, depression, and social isolation was partially supported. The emotional loneliness subscale was moderately correlated with general loneliness and depression; however, it had a less-than-weak correlation with social isolation and presence of a partner in both young and older adults. The social loneliness subscale had a moderate-to-strong correlation with general loneliness and social isolation; however, a weak or negligible correlation was observed with depression and presence of a partner in both young and older adults. The positive moderate correlation between emotional/social loneliness and general loneliness was consistent with the results of previous studies (Green et al., 2001), which supported its convergent validity. However, the negligible correlations between emotional loneliness and social isolation and between social loneliness and depression were inconsistent with the results of previous studies (Wolters et al., 2023). Nevertheless, this result may be consistent with the hypothesized construct of two types of loneliness, where emotional loneliness reflected the quality and social loneliness reflected the quantity of social connections (Gierveld, 1998). Social isolation was closely associated with several social connections, whereas depression was associated with affective social connections (Wolters et al., 2023). A possible reason for the clear distinction between the two types of loneliness could be explained by the characteristics of Japanese culture. A study revealed that Japanese individuals had lower interpersonal trust than Swedish individuals (Otsuka et al., 2015). This was attributed to the emphasis on modesty in Japanese culture. Japanese individuals tended to express emotions they believe others expect rather than their true feelings, which may contribute to low interpersonal trust even during social interactions. Future studies should explore the possible reasons for the clear distinction between social and emotional loneliness.

The fourth hypothesis that emotional loneliness would have a stronger correlation with presence of a partner and depression compared with social loneliness was partially supported. The stronger association between emotional loneliness and depression than that between social loneliness and depression was consistent with the results of a previous study (Wolters et al., 2023). However, the association between emotional and social loneliness and presence of a partner was inconsistent with those of a previous study (Green et al., 2001). We observed no significant differences in the association between emotional and social loneliness and presence of a partner in older adults. However, we observed a significant difference among young adults, although it was in the opposite direction from a previous study. Unexpectedly, the correlation between emotional/social loneliness and presence of a partner was weak or negligible in both young and older adults. A possible explanation was the historical time change between this and the previous study (Green et al., 2001). The relationship between partnership status and loneliness has changed and the association has been getting weaker over time (Böger and Huxhold, 2020). In addition, 23 years have passed since the previous study (Green et al., 2001), and the historical time change may have affected our results. Although we found a significantly stronger correlation between social loneliness and presence of a partner than between emotional loneliness and presence of a partner in the young adult sample, the correlation was weak. Therefore, whether this difference is meaningful remains questionable. This study supported the discriminant validity of the Japanese translation of the DJGLS through a stronger correlation between emotional loneliness and depression than social loneliness and depression. However, this result should be interpreted with caution since the item included in the emotional loneliness factor for the younger adult in this study was different from the original emotional loneliness factor. Furthermore, future studies should examine the association between the social and emotional loneliness and presence of a partner.

Finally, we exploratorily examined the characteristics of each item by subscales of the DJGLS via the Rasch model. In the young adult sample, the difficulty parameters ranged from −0.82 to 0.84 and −0.89 to 2.11 for emotional and social loneliness, respectively. In the older adult sample, these ranged from −1.51 to 1.45 and −1.14 to 2.06, respectively. The WMS and UMS were under 2.00 for all the items in both samples. For emotional loneliness, items covered respondents with various trait levels for both samples. However, for social loneliness, item 1 had a relatively high difficulty in both samples. This could indicate that individuals with extremely high trait levels tended to mark item 1 as yes (1). Conversely, individuals with high or low trait levels tended to mark it as no (0). This may be due to the item’s wording, which was “There is always someone I can talk to about my day-to-day problems.” Other items in the social loneliness factor asked the number of people or friends an individual trusted or felt close to. However, “someone” could include only a few people, and the person did not need to be close if the individual could talk to them regarding day-to-day problems. Thus, item 1 only captured information on individuals with extremely high trait levels, which may require further consideration of whether including it is informative for social loneliness in future studies. This finding may also reflect cultural factors specific to Japanese society, where discussing personal problems with others can be influenced by concepts such as social rigidity and relational mobility, potentially making individuals more selective about whom they consider as “someone” they can talk to about day-to-day problems.

The current study contributes to clinical practice and research on loneliness in Japan. The results of this study support the importance of examining loneliness from a multidimensional perspective. Emotional and social loneliness may function differently, with emotional loneliness being more strongly associated with psychological distress while social loneliness is more strongly associated with social isolation. Different intervention and prevention strategies are recommended for the two types of loneliness. For example, cognitive restructuring is recommended for emotional loneliness while behavioral activation is recommended for social loneliness (Walsh et al., 2025). The Japanese translation of the DJGLS demonstrates initial evidence of validity and reliability for assessing these distinct loneliness dimensions among Japanese young and older adults. This scale has the potential to facilitate more nuanced research into cultural specificities of loneliness experiences in Japan and inform the development of targeted interventions that address the unique needs of Japanese populations.

This study has several limitations. First, we did not examine the test–retest reliability. Future studies should assess the test–retest reliability of the Japanese translation of the DJGLS to ensure its temporal stability. Second, our sample only included participants aged 18–29 and 65 years or older, which left the usability of the scale for those aged 30–64 years unexplored. Future studies should examine its applicability among this middle age group. Third, the gender distribution in our sample was imbalanced across age groups. The young adult sample consisted predominantly of female participants, while the older adult sample included more male participants. Future research should investigate the association between sex and loneliness using more gender-balanced samples. Finally, this study focused exclusively on a Japanese population, which limited the generalizability of the findings to other cultural contexts. Notably, the Japanese translation of the DJGLS for young adults included different items compared with the original version, which made it unclear whether these differences were attributable to cultural variations or age-specific factors. Research examining the factor structure of loneliness among young adults is scarce, even in international contexts. Furthermore, generational changes may influence how loneliness is conceptualized. Therefore, cross-cultural studies that directly compare the Japanese and English versions of the DJGLS are essential to better understand the underlying similarities and differences in the measurement structures. Such studies would also provide insights into how loneliness manifests across different cultures and age groups and can contribute to a more nuanced understanding of this complex phenomenon.

## 5 Conclusion

This study supported the use of the DJGLS in young and older Japanese adults, providing initial evidence of the validity and reliability of the Japanese translation and the characteristics of the items in the emotional and social loneliness factors. The two-factor structure of emotional and social loneliness was supported across both age groups, with differential associations observed between the loneliness dimensions and related constructs. The distinct associations of emotional and social loneliness with depression and social isolation may encourage future studies to examine loneliness as two separate types rather than a general factor. The Japanese translation of the DJGLS shows promise as a tool for assessing the dimensions of loneliness in Japanese populations and may contribute to culturally informed research and interventions.

## Data Availability

Detailed data are available from the corresponding author upon reasonable request.

## Footnotes

^1^The original research protocol comprised an analysis to examine the measurement invariance of the DJGLS between young and older adults. However, we did not examine the measurement invariance owing to the difference in an item in emotional loneliness factor as a result of the confirmatory factor analysis.

^2^ https://doi.org/10.17605/OSF.IO/JECA6

^3^Although Koğar and Koğar (2023) suggested the use of a unidimensional loneliness factor of the DJGLS from a bifactor model, we also conducted bifactor exploratory structural equation modeling (ESEM) as a supplemental analysis in case bi-factor ESEM would indicate two clearly distinct factors. The results are shown in the Supplementary file. Consistent with Koğar and Koğar (2023) and contrary to our aim, bi-factor ESEM did not clearly support two distinct factors of emotional and social loneliness. Therefore, we accepted the two-correlated factor model in this study.
