# Supplementary material for: Psychometric properties of the Japanese translation of the De Jong Gierveld Loneliness Scale for young and older adults
Source: Front Psychol. 2025 Jun 27;16:1542961. doi: 10.3389/fpsyg.2025.1542961 (PMC12245850; doi:10.3389/fpsyg.2025.1542961)
Supplement: Supplementary file 1 [file Table_1.docx]

Supplemental Table The result of bi-factor exploratory structural equation models for young and older adults

|  | Young adults | | | Older adults | | |
| --- | --- | --- | --- | --- | --- | --- |
|  | General | Social Loneliness | Emotional Loneliness | General | Social Loneliness | Emotional Loneliness |
| Item 1 | .11 | **.86** | .19 | .59 | **.47** | .00 |
| Item 2 | .75 | .11 | **.24** | .66 | -.34 | **.54** |
| Item 3 | .75 | .05 | **.33** | .44 | .03 | **.67** |
| Item 4 | .25 | **.91** | .04 | .82 | **.31** | -.09 |
| Item 5 | .71 | -.49 | **-.01** | .29 | -.09 | **.69** |
| Item 6 | .79 | -.09 | **.10** | .55 | -.17 | **.62** |
| Item 7 | .21 | **.86** | -.07 | .94 | **.00** | -.21 |
| Item 8 | .35 | **.86** | -.06 | .95 | **.06** | -.19 |
| Item 9 | .78 | -.13 | **.35** | .42 | .00 | **.70** |
| Item 10 | .59 | -.00 | **.83** | .36 | .17 | **.76** |
| Item 11 | .23 | **.86** | -.01 | .80 | **.29** | -.04 |
|  |  |  |  |  |  |  |
| Model Fit |  |  |  |  |  |  |
| CFI | .99 |  |  | .99 |  |  |
| RMSEA | .04 |  |  | .03 |  |  |
| SRMR | .02 |  |  | .03 |  |  |

*Note.* Factor loadings for items on each subscale are shown in bold.
